# NLRP3 inflammasome activation results in liver inflammation and fibrosis in mice infected with *Schistosoma japonicum* in a Syk-dependent manner

**DOI:** 10.1038/s41598-017-08689-1

**Published:** 2017-08-14

**Authors:** Ya-Qi Lu, Shan Zhong, Nan Meng, Yin-Ping Fan, Wang-Xian Tang

**Affiliations:** Institute of Liver Diseases, Tongji Hospital of Tongji Medical College, Huazhong University of Science and Technology, 1095 Jie-Fang Avenue, Wuhan, 430030 Hubei Province People’s Republic of China

## Abstract

Granulomatous and fibrosing inflammation in response to soluble egg antigen (SEA) from *Schistosoma japonicum* (*S. japonicum*) is the main pathological process of *S. japonicum* infection. Inflammasome activation has recently been implicated in the pathogenesis of liver disease. However, the role of inflammasome activation in schistosomiasis-associated liver fibrosis (SSLF) has not been extensively studied. In this study, it is demonstrated that the NLRP3 inflammasome is markedly activated in mouse HSCs both *in vivo* and *in vitro* during *S. japonicum* infection. Furthermore, it is demonstrated that inhibition of NLRP3 inflammasome significantly alleviates the liver inflammation and collagen deposition that are induced by infection with *S. japonicum*. The mechanism of SEA-induced NLRP3 inflammasome activation is studied in isolated, cultured mouse HSCs and it is shown that SEA-induced NLRP3 inflammasome activation in HSCs is dependent upon the activities of spleen tyrosine kinase (Syk), an enzyme usually associated with a pathogen recognition receptor for fungal pathogens. Moreover, it is demonstrated that Dectin-1 and JNK signaling are also involved in SEA-induced NLRP3 inflammasome activation in HSCs. These data shed new light on the mechanisms of NLRP3 inflammasome activation during an infection with *S. japonicum*, and further characterize its role in schistosomiasis-associated liver fibrosis (SSLF).

## Introduction

Schistosomiasis is a neglected tropical disease caused by parasitic flukes of the genus *Schistosoma*. It represents a serious public health problem worldwide^1, 2^. According to WHO statistics, there were almost 240 million people worldwide infected with the various types of schistosomiasis in 2016, and more than 700 million people were living in endemic areas are at risk of infection^3^. *Schistosoma japonicum* (*S. japonicum*), is one of the three main species of schistosome responsible for human infections, and is the only human blood fluke that occurs in China^4, 5^. Once a person is infected with *S. japonicum*, the cercariae penetrate the human skin, transform into schistosomula and migrate through the circulatory system to reach the portal system^6^. Here they mature into adult worms and lay eggs. These eggs are pathogenic to the human host because they induce granulomatous inflammation and tissue damage^7, 8^. Because the human immune response is unable to clear the parasitic infection, the liver is subjected to ongoing cycles of focal inflammation and healing that lead to vascular obstruction and tissue fibrosis^1^. Since granulomatous and fibrosing inflammation in the liver constitute the main pathology during *S. japonicum* infection^9^, understanding the immunopathogenisis of granulomatous inflammation may elucidate novel therapeutic targets and approaches by which to treat schistosomiasis-associated liver fibrosis (SSLF).

Recently, several studies have suggested that inflammasome, a multiprotein oligomer that is one of the components of the innate immune system, is involved in the pathogenesis of the inflammatory response via the processing of caspase-1 and IL-1*β* to an active stage following human liver disease^10–14^. Moreover, the present authors have recently demonstrated that the activation of caspase-1 could be dependent on the NLRP3 inflammasome during *S. japonicum* infection. The NLRP3 inflammasome is the best-characterized inflammasome to date, as it can be efficiently stimulated by a diverse range of pathogen-associated molecular patterns (PAMPs), danger-associated molecular patterns (DAMPs) and microbial invasion^15–17^. It is an intracellular multiprotein complex composed of the adaptor molecule apoptosis-associated speck-like protein containing a CARD (ASC), and procaspase-1. NLRP3 serves as a protein complex scaffold, and ASC bridges NRLP3 to the effector protein procaspase-1, causing its activation, which in turn cleaves the precursor forms of IL-1β and IL-18 to their bioactive forms^18^. Three molecular models have been put forward to explain the mechanisms lying behind NLRP3 inflammasome activation, including lysosomal activity, ion fluxes, particularly potassium (K+) efflux and reactive oxygen species (ROS)^19, 20^. Although the molecular mechanisms of NLRP3 inflammasome activation are still being determined, recent studies have identified an upstream kinase, spleen tyrosine kinase (Syk), as a critical mediator of the process. Syk is a cytoplasmic protein-tyrosine kinase and a member of the Src family of non-receptor tyrosine kinases. It plays a vital role in transmitting signals from a variety of cell surface receptors, such as CR3, FcγR, Dectin-1 and apoptotic cell-recognizing receptor^21, 22^. Many studies have demonstrated that Syk can be coupled to NLRP3 inflammasome to activate caspase-1 for anti-fungal immunity^23, 24^. Moreover, several recent studies have demonstrated that Syk also plays a critical role in NLRP3 inflammasome activation during *Malarial Hemozoin* infection^25^ as well as during *Chlamydial* ^21^ and *Mycobacterium abscessus* infections^26^. It has further been shown that Syk can regulate ROS production^27, 28^ and lysosomal activity^25, 29^, two of the major signals for NLRP3 inflammasome activation. Meanwhile, previous studies by the present authors have found that *in vitro* activation of NLRP3 inflammasome in cultured mouse hepatic stellate cells (HSCs) is dependent on ROS production and lysosomal activity during *S. japonicum* infection. Therefore, it is hypothesized that Syk signaling may also be required for NLRP3-dependent caspase-1 activation during *S. japonicum* infection.

Our previous study has demonstrated that *S. japonicum* infection *in vivo* or soluble egg antigen (SEA) stimulation *in vitro* can activate the NLRP3 inflammasome in HSCs^11^. Moreover, the mechanisms of NLRP3 inflammasome activation in HSCs during *S. japonicum* infection were shown to be dependent on ROS production and lysosomal activity^11^. In the present study, we aimed to further investigate the role of NLRP3 inflammasome activation in liver fibrogenesis during *S. japonicum* infection, and to determine whether NLRP3 inflammasome activation in HSCs is dependent on Syk kinase signaling.

## Results

### Verification of vector insertion in liver tissues

**Figure 1:**
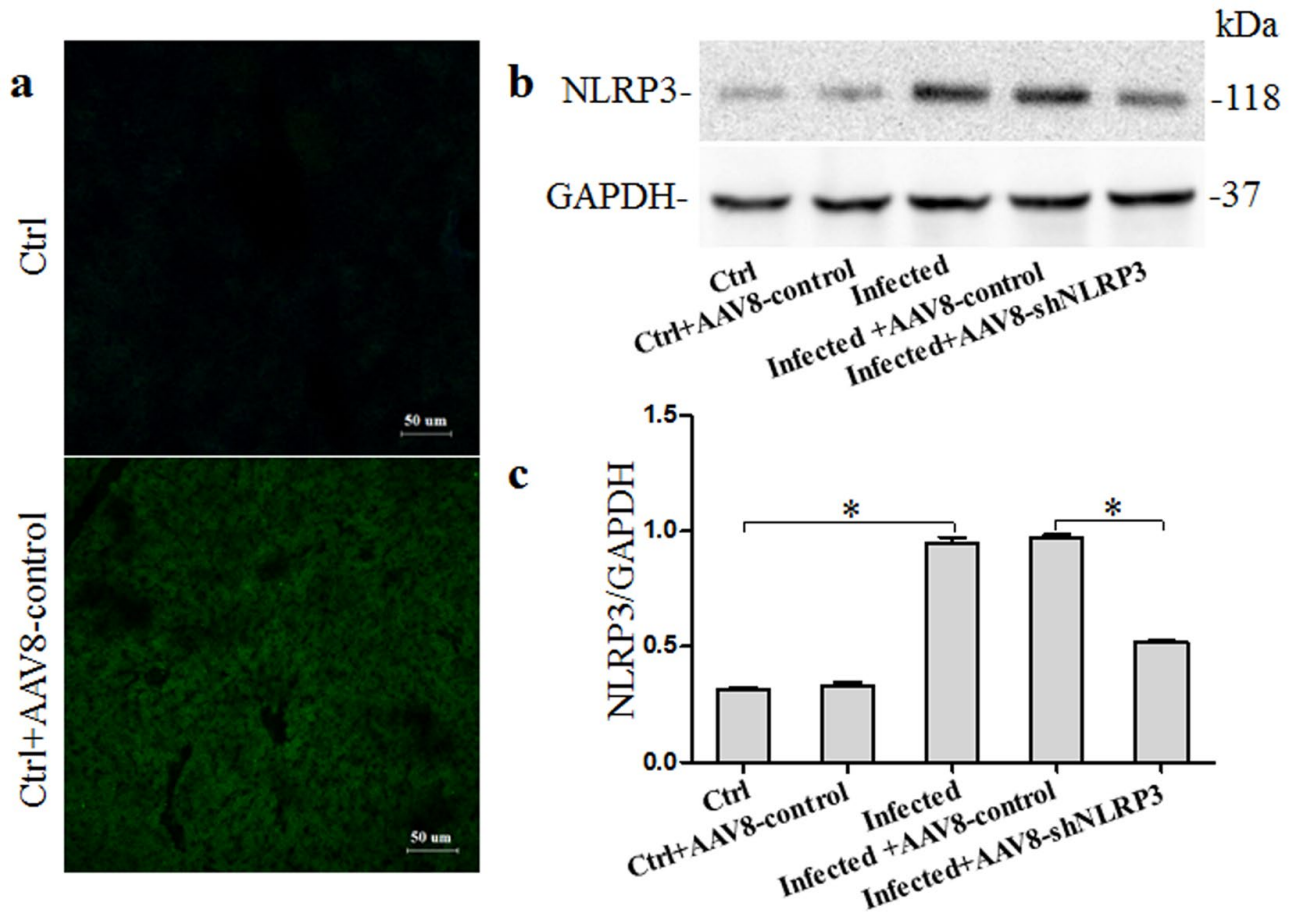
Verification of AAV8 insertion and inhibition of NLRP3 in the liver tissues of mice infected wth *S. Japonicum*: (**a**) representative confocal fluorescence image showing GFP expression in the liver; (**b**) western blot gels and (**c**) summarized data showing the expression of NLRP3, full-length blots are included in the Supplementary Fig. S1. Ctrl: control (n = 4); Ctrl + AAV8-control: control mice injected with AAV8-control which express GFP (n = 4); Infected: mice infected with *S. Japonicum* (n = 6); Infected + AAV8-control: Infected mice injected with AAV8-control (5 × 10^9^ vg per animal) (n = 6); Infected + AAV8-shNLRP3: Infected mice injected with AAV8-shNLRP3 (5 × 10^9^ vg per animal) (n = 6). Results are a representative of at least three independent experiments. ^*^p < 0.05.

The presence of green fluorescent protein (GFP) in liver tissue was detected to demonstrate the achievement of infection and vector insertion via confocal laser scanning microscopy. After 6 weeks of infection with *S. japonicum* cercariae and adeno-associated virus AAV8, green fluorescent signals from GFP were observed in the liver tissues of mice infected with AAV8-control (Fig. 1a). Western blot analysis demonstrated that the protein expression levels of NLRP3 were decreased in mice infected with AAV8–shNLRP3 compared to mice infected with AAV8-control (Fig. 1b,c and Supplementary Fig. S1). Collectively, the results demonstrated the successful vector insertion of AAV8–shNLRP3, and showed that AAV8–shNLRP3 successfully inhibited protein expression levels of NLRP3 *in vivo*.

### Mouse model of schistosomiasis-associated liver fibrosis (SSLF)

**Figure 2:**
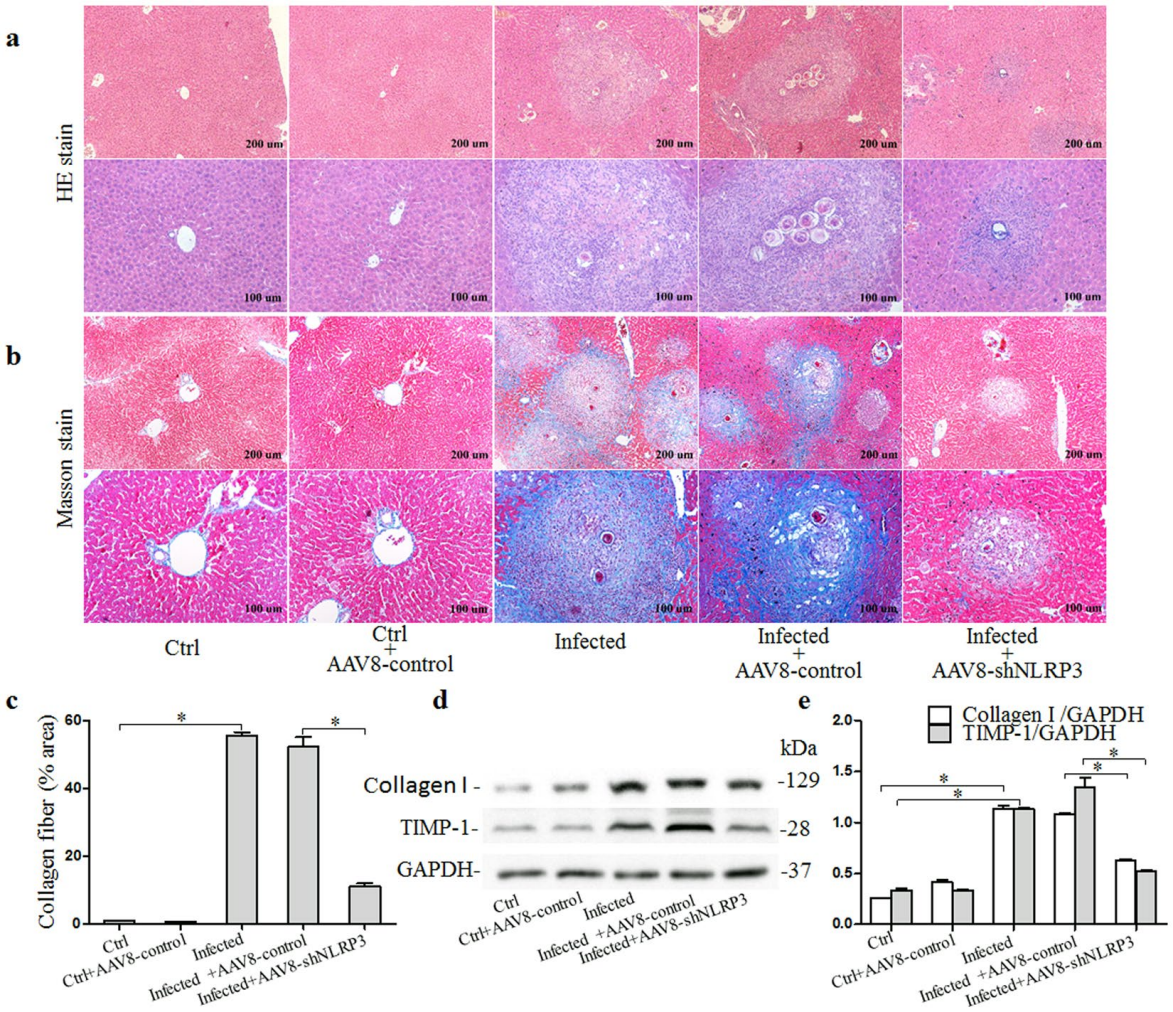
Pathological changes in liver tissues from mice 6 weeks after infection with *S. Japonicum*: (**a**) Representative images of HE stained liver tissue; (**b**) Representative images of masson stained liver tissue and (**c**) quantitative analysis of positive stain of collagen fiber; (**d**) western blot gels and (**e**) summarized data showing the expression of collagen-1 and TIMP-1, full-length blots are included in the Supplementary Fig. S2. Ctrl: control (n = 4); Ctrl + AAV8-control: control mice injected with AAV8-control (n = 4); Infected: mice infected with *S. Japonicum* (n = 6); Infected + AAV8-control: Infected mice injected with AAV8-control (5 × 10^9^ vg per animal) (n = 6); Infected + AAV8-shNLRP3: Infected mice injected with AAV8-shNLRP3 (5 × 10^9^ vg per animal) (n = 6). Results are a representative of at least three independent experiments. ^*^p < 0.05.

After 6 weeks of infection with *S. japonicum* cercariae, the Masson trichome stained mouse liver tissue samples were found to exhibit significant collagen deposition at the periphery of the eosinophilic granuloma, while *S. japonicum* eggs remained in the liver portal vein. These characteristics of SSLF were not observed in the livers of control mice (Fig. 2a,b). The results suggest that the SSLF model had been successfully established in *S. japonicum*-infected mice. As shown in Fig. 2b,c, more collagen was deposited in the livers of *S. japonicum*-infected mice compared to those of control mice. Further, the *S. japonicum*-induced increases in collagen deposition were markedly reduced in mice that had been pretreated with the AAV8-shNLRP3 vector. It was also demonstrated that the increased expression of the proteins collagen I and metalloproteinase inhibitor-1 precursor (TIMP-1) after infection with *S. japonicum* was significantly attenuated in mice infected with AAV8-shNLRP3 (Fig. 2d,e and Supplementary Fig. S2).

### NLRP3 inflammasome is activated in HSCs in the livers of mice infected with *S. japonicum*

**Figure 3:**
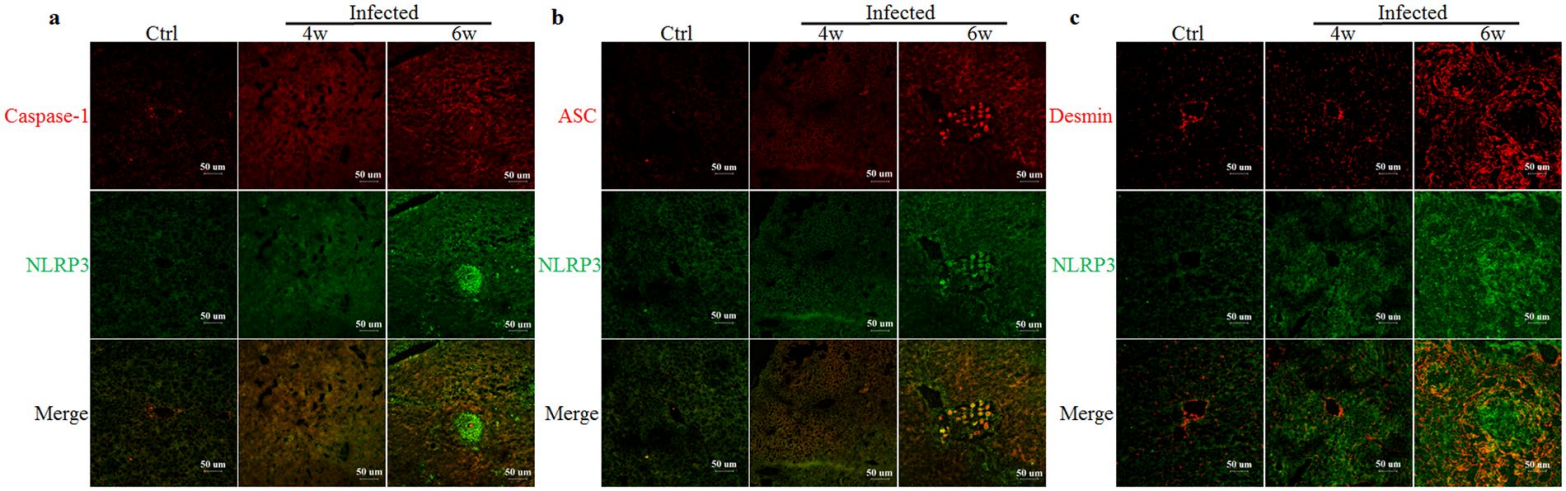
Confocal fluorescence images of NLRP3 inflammasome formation in the livers of mice infected with *S. japonicum*: the co-localization of NLRP3 with caspase-1 (**a**), ASC (**b**) and desmin (**c**). Ctrl: control (n = 4); 4w: mice infected with *S. Japonicum* for 4 weeks (n = 6); 6w: mice infected with *S. japonicum* for 6 weeks (n = 6). Results are a representative of at least three independent experiments.

Using confocal microscopy, the formation of NLRP3 inflammasome in the liver of mice infected with *S. japonicum* was investigated. As shown in Fig. 3a,b, the co-localization of NLRP3 with ASC or caspase-1 was increased in the liver tissue of mice infected with *S. japonicum* compared to control mice. Such co-localization suggests increased formation of NLRP3 inflammasome in liver tissues of the infected mice. To demonstrate that NLRP3 inflammasome was also formed in HSCs in the liver of these infected mice, we examined the co-localization of NLRP3 with the HSC marker desmin. As shown in Fig. 3c, co-localization of NLRP3 with desmin was increased around liver sinusoids and *S. japonicum* eggs in the portal area of *S. japonicum* infected mice compared with control mice. These results collectively demonstrate the formation and activation of NLRP3 inflammasome in HSCs in the mouse liver during *S. japonicum* infection.

### SEA induces NLRP3 inflammasome activation in HSCs

**Figure 4:**
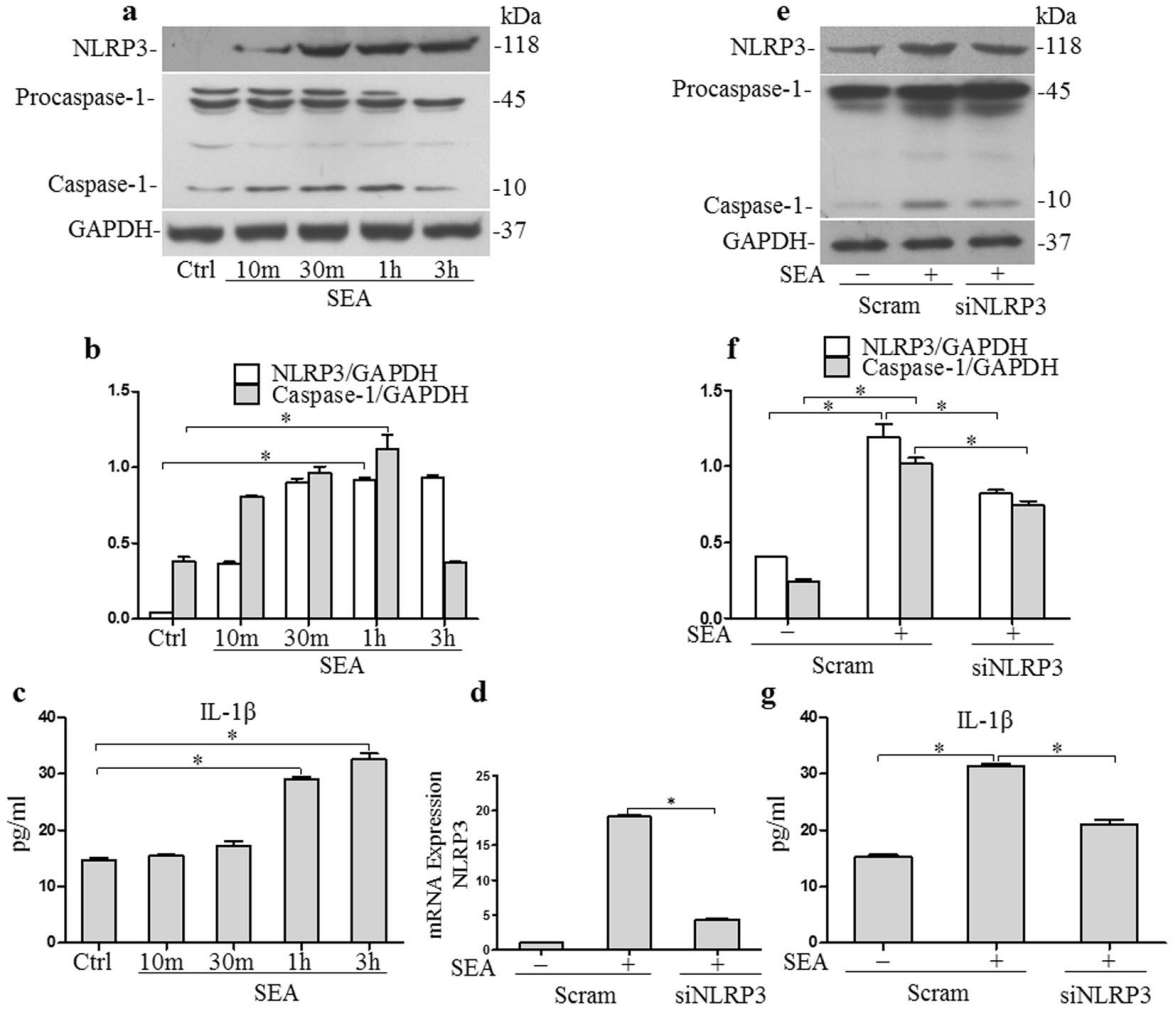
SEA induces NLRP3 inflammasome activation in HSCs: (**a**) Western blot gels, (**b**) summarized data showing the expression of NLRP3, pro-caspase-1 and activated caspase-1 in HSCs after stimulation with SEA (50 μg/ml), full-length blots are included in the Supplementary Fig. S3, and (**c**) IL-1β production in cell supernatant; (**d**) Summarized relative mRNA levels of NLRP3 in HSCs in the presence or absence siNLRP3, as determined by RT-PCR. HSCs were incubated with or without SEA (50 μg/ml) for 2 h in the presence or absence siNLRP3; (**e**) Western blot gels, (**f**) summarized data showing the expression of NLRP3, pro-caspase-1, activated caspase-1 in HSCs, full-length blots are included in the Supplementary Fig. S4; (**g**) IL-1β production in cell supernatant. Scram: scrambled siRNA; siNLRP3: NLRP3 siRNA. Results are a representative of at least three independent experiments. ^*^p < 0.05.

The activation of caspase-1 and IL-1β production induced by SEA in infected HSCs was measured at various time points after the start infection. As can be seen from the results (Fig. 4a–c and Supplementary Fig. S3), SEA induced caspase-1 activation and IL-1β production in infected HSCs in a time dependent mannar. To confirm that caspase-1 activation was dependent on NLRP3 inflammasome activation, we silenced NLRP3 gene expression by RNA interference. When compared with cells treated with non-target small interference RNA (siRNA), cells treated with siNLRP3 (a small interfering RNA designed to knock down the expression of NLRP3) showed apparent reduction in mRNA expression as well as significantly lower protein expression (Fig. 4d,e and Supplementary Fig. S4). Infected HSCs were then treated with SEA (50 μg/ml) for 2 h, and it was found that the activation of caspase-1 and IL-1β production in cells treated with siNLRP3 was reduced (Fig. 4e–g and Supplementary Fig. S4). These results suggest that SEA induced activation of caspase-1 and IL-1β production are dependent on expression of NLRP3 inflammasome in infected HSCs.

### NLRP3 silencing blocks fibrotic changes in infected HSCs induced by SEA *in vitro*

**Figure 5:**
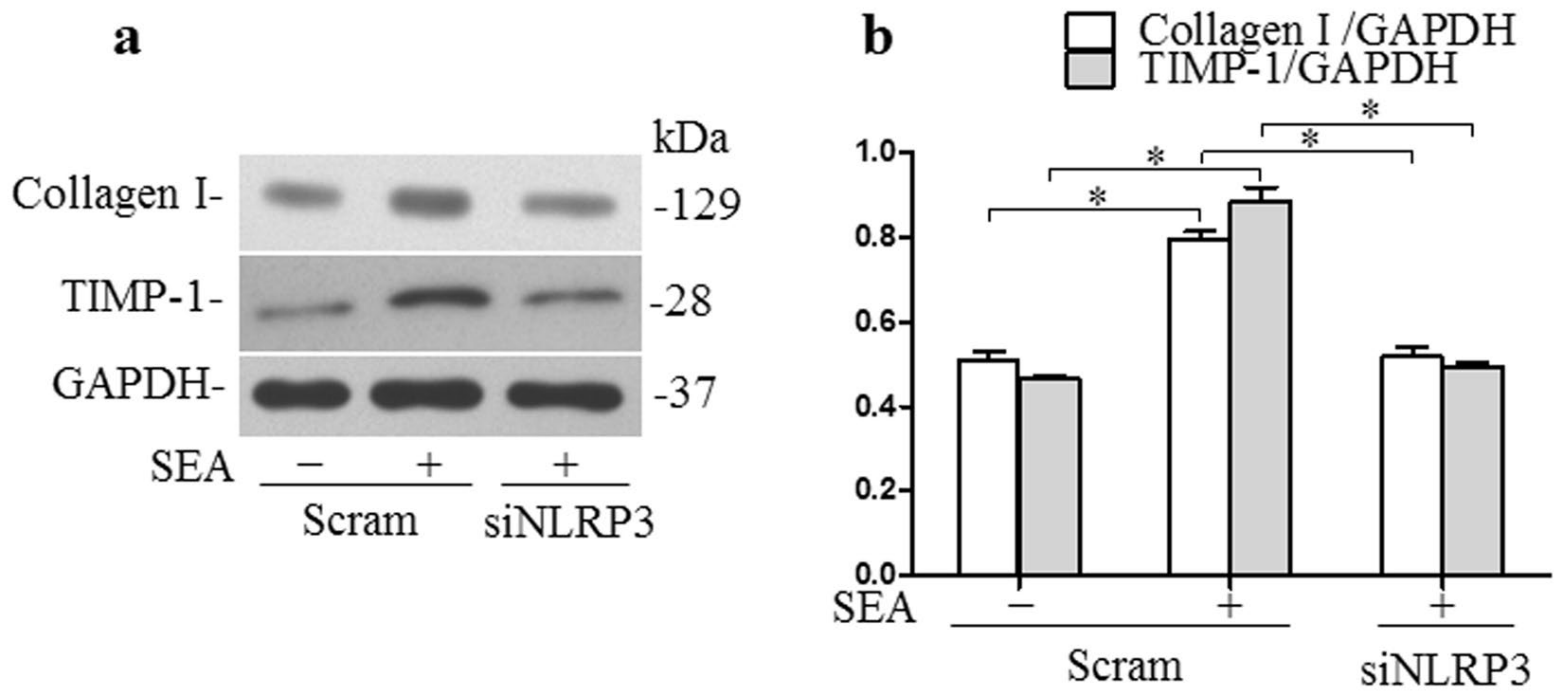
NLRP3 silencing blocks fibrotic changes induced by SEA *in vitro*: (**a**) Western blot gels and (**b**) summarized data showing the expression of collagen-1, TIMP-1 and GAPDH in HSCs incubated with or without SEA (50 μg/ml) for 24 h in the presence or absence siNLRP3, full-length blots are included in the Supplementary Fig. S5. Results are a representative of at least three independent experiments. ^*^p < 0.05.

Given that the above results suggested that SEA can induce NLRP3 inflammasome formation and activation, we subsequently investigated whether NLRP3 inflammasome activation in HSCs was involved in the SEA-induced fibrogenic phenotype change of infected HSCs *in vitro*. We transfected the infected HSCs with NLRP3 siRNA, and then treated the resulting cells with SEA (50 μg/ml) for 24 h. The HSC protein lysates were then analyzed by Western blot. As shown in Fig. 5a,b and Supplementary Fig. S5, the expression of collagen I and TIMP-1 protein was found to be increased after induction of infected HSCs with SEA, and was significantly attenuated when these cells were transfected with NLRP3 siRNA.

### SEA induced NLRP3 inflammasome activation depending on Syk signaling

**Figure 6:**
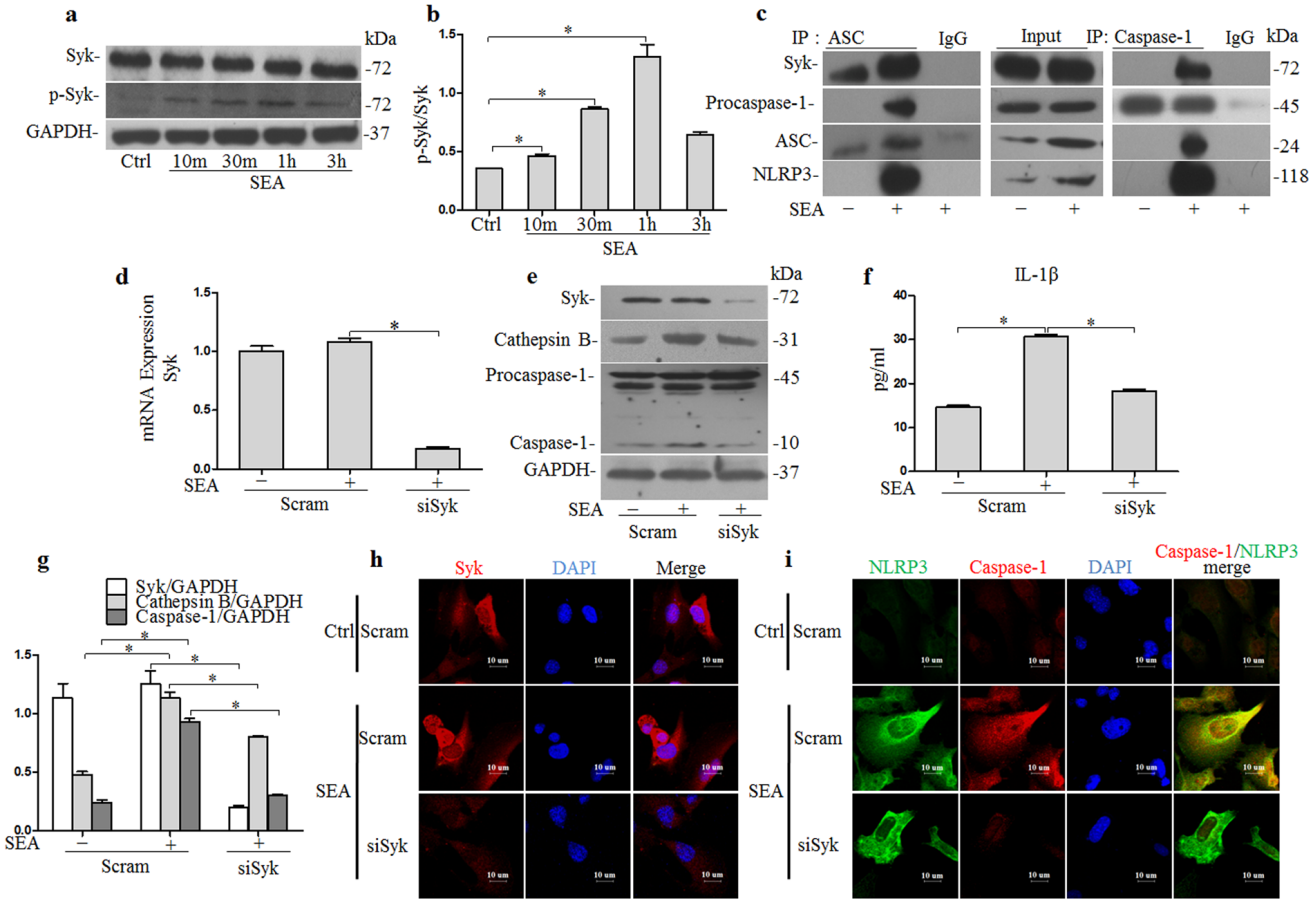
Involvement of Syk in NLRP3 inflammasome activation by connecting to components of the NLRP3 inflammasome and modulating cathepsin B activation: (**a**) Western blot gels and (**b**) summarized data showing the expression of Syk, p-Syk (Y525/526) and GAPDH in HSCs after stimulation with SEA (50 μg/ml), full-length blots are included in the Supplementary Fig. S6; (**c**) Immunoblot analysis of Syk, procaspase-1, ASC and NLRP3 in HSCs incubated with or without SEA (50 μg/ml) for 2 h (middle image), after IP with anti-ASC antibody (left hand image), and anti-caspase-1 antibody (right hand image). Full-length blots are included in the Supplementary Fig. S7. (**d**) Summarized relative mRNA levels of Syk in HSCs in the presence or absence siSyk, as determined by RT-PCR; (**e**) Western blot gels and (**g**) summarized data showing the expression of Syk, Cathepsin B, pro-caspase-1, activated caspase-1 and GAPDH in HSCs incubated with or without SEA (50 μg/ml) for 2 h in the presence or absence of siSyk, full-length blots are included in the Supplementary Fig. S8, (**f**) IL-1β production in cell supernatant. Representative confocal fluorescence images show. (**h**) Syk expression and (i) co-localization of NLRP3 with caspase-1 in HSCs incubated with or without SEA (50 μg/ml) for 2 h in the presence or absence siSyk. SiSyk: Syk siRNA. Results are a representative of at least three independent experiments. ^*^p < 0.05.

Our previous research had demonstrated that SEA induced caspase-1 activation and IL-1β production in an NLRP3 inflammasome-dependent manner that required the presence of active cathepsin B, and that the widely expressed Syk was shown to be required for cathepsin B release into the cytosol. We therefore investigated whether Syk signaling was required for NLRP3 inflammasome-dependent caspase-1 activation and IL-1β production following SEA infection. As shown in Fig. 6a,b and Supplementary Fig. S6, the levels of phosphorylated Syk were markedly increased after SEA treatment, and peaked at 1 h post-treatment. To examine whether Syk can modulate the formation of the NLRP3 inflammasome by directly interacting with its components, we immunoprecipitated either caspase-1 or ASC (as important constituents of NLRP3 inflammasome) and then immunoblotted the precipitates in order to identify potential protein interaction partners associated with it by western blotting analysis. The results showed that caspase-1 and ASC both strongly associated with Syk upon stimulation with SEA (Fig. 6c and Supplementary Fig. S7). We subsequently investigated the effect of inhibition of Syk on caspase-1 activation and IL-1β production, and found that either silencing of Syk by specific siRNA transfection (Fig. 6d–g and Supplementary Fig. S8), or pharmacological inhibition using piceatannol (Supplementary Figs S9 and S10), significantly inhibited caspase-1 activation and IL-1β production in response to SEA treatment in infected HSCs. This indicated that Syk activation was essential for caspase-1 activation and IL-1β production in HSCs infected with SEA. Similarly, we demonstrated that silencing of the Syk gene markedly inhibited SEA-induced co-localization of NLRP3 with caspase-1 (Fig. 6h,i). Moreover, this inhibition of Syk also reduced the secretion of activated cathepsin B induced by SEA treatment in infected HSCs (Fig. 6e,g).

### Dectin-1 plays an essential role in Syk-dependent NLRP3 inflammasome activation following infection with SEA

**Figure 7:**
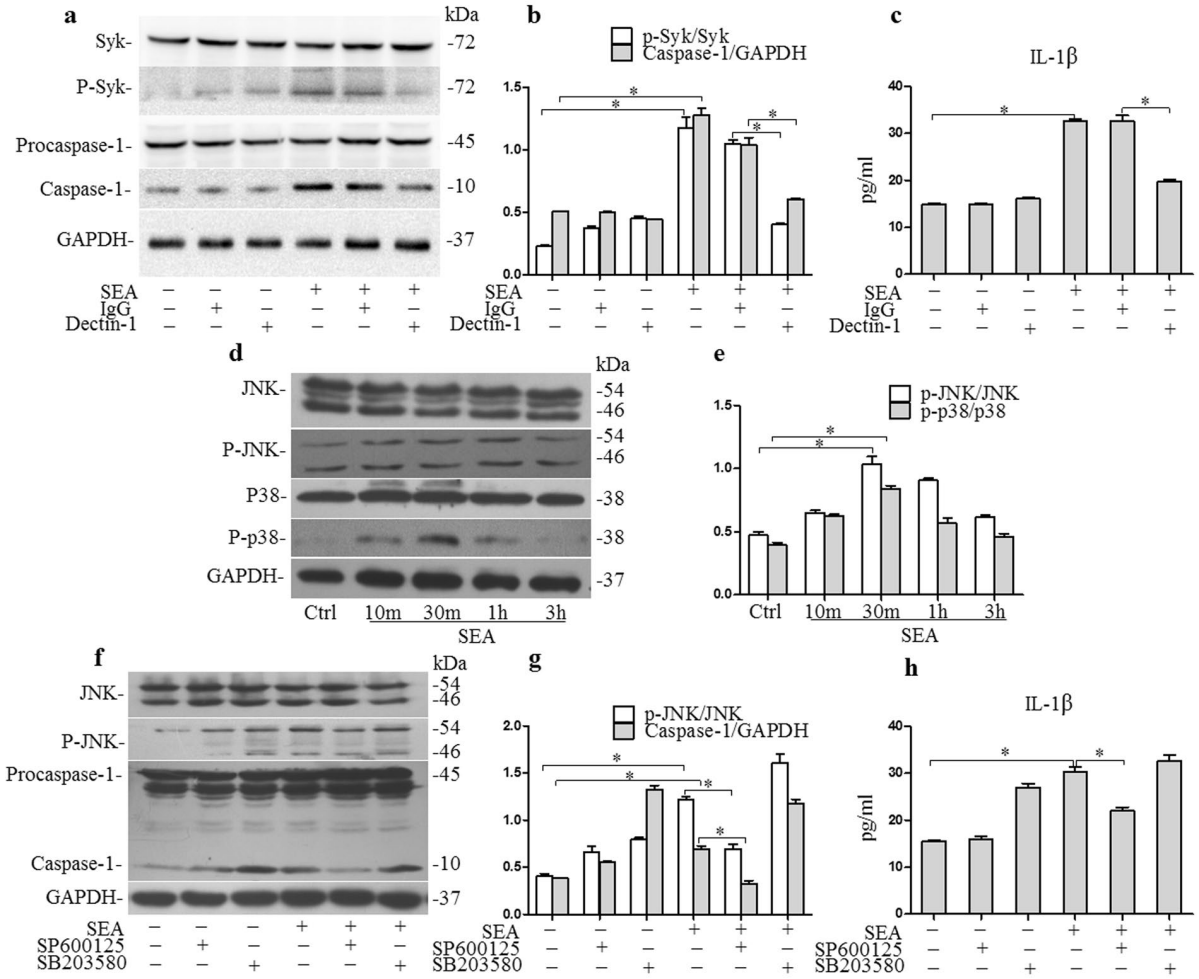
Dectin-1 and JNK are involved in NLRP3 inflammasome activation: (**a**) western blot gels and (**b**) summarized data showing the expression of Syk, p-Syk (Y525/526), procaspase-1, activated caspase-1 and GAPDH in HSCs incubated with or without SEA (50 μg/ml) in the presence or absence anti-Dectin-1 antibody, full-length blots are included in the Supplementary Fig. S11, (**c**) IL-1β production in cell supernatant; (**d**) Western blot gels and (**e**) summarized data showing the expression of JNK, p-JNK (Thr 183 + Tyr 185), p38, p-p38 and GAPDH in HSCs after stimulation with SEA (50 μg/ml), full-length blots are included in the Supplementary Fig. S12; (**f**) Western blot gels and (**g**) summarized data showing the expression of JNK, p-JNK (Thr 183 + Tyr 185), procaspase-1, activated caspase-1 and GAPDH in HSCs incubated with or without SEA (50 μg/ml) for 2 h in the presence or absence inhibitors for JNK (SP600125) and p38 (SB 203580), full-length blots are included in the Supplementary Fig. S13, (**h**) IL-1β production in cell supernatant. Results are a representative of at least three independent experiments. ^*^p < 0.05.

The previous experimental results had demonstrated that NLRP3 inflammasome activation was dependent on Syk signaling. Given that Syk is known to play a key role in transmitting signals from a variety of cell surface receptors, including the C-type lectin receptor Dectin-1, and given that a recent study has found that Dectin-1 is involved in *C. albicans*-induced inflammasome activation^30^, we sought to determine whether Dectin-1 was also involved in SEA-induced NLRP3 inflammasome activation in infected HSCs. By treating HSCs with the Dectin-1 primary antibody for 1 h before a 2 h incubation with SEA (50 μg/ml) to inhibit Dectin-1, we assessed the activation of caspase-1 by immunoblot analysis and IL-1β production by ELISA and found that the increase in activated caspase-1 and IL-1β production that had been induced by SEA was successfully reduced in HSCs that had been pre-treated with the primary antibody of Dectin-1 (Fig. 7a–c and Supplementary Fig. S11).

### JNK is involved in SEA induced NLRP3 inflammasome activation

As shown in Fig. 7d,e and Supplementary Fig. S12, stimulation of infected HSCs with SEA triggered the activation of both JNK and p38, phosphorylated JNK and p38 were increased as early as 10 min after treatment of the HSCs with SEA (50 μg/ml). To verify whether JNK and p38 were also involved in the process of NLRP3 inflammasome activation, JNK and p38 were silenced using special pharmacological inhibitors for JNK (SP600125) and p38 (SB 203580). As shown in Fig. 7f–h and Supplementary Fig. S13, inhibition of JNK in HSCs stimulated with SEA resulted in a significant reduction in activated caspase-1 production and IL-1β production, but inhibition of p38 produced no such reduction and in fact resulted in a slight increase in production of activated caspase-1 and IL-1β production. These results indicate that JNK is involved in SEA induced NLRP3 inflammasome activation in infected HSCs.

## Discussion

Chronic inflammation and inflammasome activation are significant factors in the pathogenesis of liver fibrosis^10, 12, 31^. Because it can be efficiently stimulated by a diverse range of factors, the NLRP3 inflammasome is one of the most-studied inflammasomes to date with respect to its role in antibacterial responses^26, 32^, antifungal responses^33^ and in the pathology of various human diseases^34, 35^. In the present study, it has been demonstrated that the NLRP3 inflammasome can be activated, *in vivo*, by *S. japonicum* infection and, *in vitro*, by *S. japonicum* egg antigen extracts. Inhibition of NLRP3 inflammasome successfully reduced the expression of collagen I and TIMP-1 proteins after *S. japonicum* infection both *in vivo* and *in vitro*. In addition to ROS production and lysosomal activity as potential molecular mechanisms by which the NLRP3 inflammasome is activated, we further identified an upstream protein signaling pathway, Syk, that appeared to be involved in the regulation of SEA-induced NLRP3 inflammasome activation *in vitro*.

HSCs are considered to be the main fibrogenic cell type of the liver. In response to damage which may be from a variety of sources, HSCs undergo a well-characterized activation process leading to increased expression of TIMP-1 and extracellular matrix (ECM) proteins such as collagen I^36^. It has been reported that the abnormal activation and consequent proliferation of HSCs along with disturbed ECM metabolism are critical factors in the initiation and development of liver fibrosis including SSLF^37^. The NLRP3 inflammasome has been reported to amplify chronic liver inflammation and activate HSCs^12^. Using confocal fluorescent microscopy, we identified the co-localization of NLRP3 with ASC or caspase-1, and the co-localization of NLRP3 with the HSC marker desmin were increased in liver tissues of mice infected with *S. japonicum* compared with that in normal mice, demonstrating that *S. japonicum* induced NLRP3 inflammasome formation and activation in HSCs *in vivo*. Moreover, confocal fluorescent microscopy and immunoprecipitation techniques demonstrated that SEA induced NLRP3 inflammasome formation and activation in infected HSCs *in vitro*. In other research, NLRP3 inflammsome deficiency has been associated with protection against carbon tetrachloride or thioacetamide (TAA)-induced liver fibrosis, and has been found to reduce mortality and liver injury after acetaminophen administration^38, 39^. Using NLRP3 knockout mice, it has also been reported that NLRP3 inflammasome activation was required for fibrosis development in non-alcoholic fatty liver disease (NAFLD)^40^. In the present study, the inhibition of NLRP3 inflammasome by infection with AAV8-shNLRP3, successfully attenuated collagen deposition and increased expression of TIMP-1 in the liver that had been induced by *S. japonicum* infection. In HSCs, the inhibition of NLRP3 inflammasome was also found to reduce the expression of collagen I and TIMP-1 proteins which had been increased after treatment of HSCs with SEA. It has thus been demonstrated that NLRP3 inflammasome activation is required for schistosomiasis-associated liver fibrosis development in mice.

NLRP3 inflammasome is activated by various heterogeneous environmental irritants including extracellular ATP, monosodium urate (MSU), hyaluronan, and certain exogenous stimuli such as, asbestos, silica, aluminum adjuvant, and microbial toxins^33, 41^. Even though NLRP3 inflammasome has been studied in the context of various human diseases over the course of several decades, the precise mechanisms that lead to NLRP3 inflammasome activation have not previously been fully elucidated. The three molecular mechanisms thought to be involved in NLRP3 inflammasome activation include: increased generation of ROS; enhanced lysosome membrane permeability, and; elevated K^+^ efflux^11, 24, 33^. A previous study by the present authors demonstrated that the increased generation of ROS, and enhanced lysosome membrane permeability are both factors that are involved in SEA-induced NLRP3 inflammasome activation in HSCs^11^. The role of Syk in immune responses has been investigated extensively over the past decade, and its role in the activation of the NLRP3 inflammasome has attracted particular research attention recently^42^. It was recently shown that Syk, operating downstream of the fungal pattern recognition receptor Dectin-1, controls both pro–IL-1β synthesis and inflammasome activation after cell stimulation with *C. albicans* ^23^. In the present study we have further found that SEA triggers Syk phosphorylation in HSCs. The inhibition of Syk with either piceatannol, or a Syk-specific siRNA, reduced caspase-1 activation and IL-1β production that had been induced by SEA. SEA-induced NLRP3 inflammasome activation is thus dependent on tyrosine kinase Syk in HSCs. An interesting question that remains is how tyrosine kinase Syk regulates the activation of the NLRP3 inflammasome. Previous studies have revealed that Syk and JNK influence the activity of ASC-containing inflammasomes in macrophages via a mechanism that regulates the formation of ASC specks^42, 43^. The phosphorylation of ASC during inflammasome activation was required for activation of the NLRP3 inflammasome^43^. A separate study showed that Syk could interact with ASC, and then with NLRP3 via ASC. Syk has been shown to modulate ASC pyrin domain phosphorylation by interacting with ASC^25^. The results of the present study have shown that immunoprecipitation of either caspase-1 or ASC (which are important constituent components of the NLRP3 inflammasome) was associated with Syk. These findings suggest that caspase-1 and ASC strongly associate with this kinase upon stimulation with SEA. Another possible mechanism whereby Syk can modulate NLRP3 inflammasome activation is by the modulation of ROS production or cathepsin B activation. In other research, Syk has been found to control the activation of cathepsin B, and Hz-induced IL-1β production was shown to be dependent on cathepsin B activation. The inhibition of Syk was shown to block the Hz-induced cathepsin B activation^25^. Furthermore, cathepsin B activation has been demonstrated to be involved in SEA-induced NLRP3 inflammasome activation in HSCs in a previous study by the current authors. In the present study, we found that the inhibition of Syk blocked SEA-induced cathepsin B activation.

Dectin-1, a C-type lectin-like pattern-recognition receptor has recently been shown to be involved in the activation of inflammasomes in antifungal infections, including *Candida albicans* and *Aspergillus fumigates* ^24, 30, 44^. Dectin-1 contains an immunoreceptor tyrosine-based activation motif-like motif (ITAM) in its cytoplasmic tail. Upon ligand binding, Syk tyrosine kinase is recruited to the Dectin-1 receptor complex through ITAM interaction and is activated^45^. Studies have shown that both Dectin-1 and Syk are essential for IL-1β transcription activated by β-glucans in human macrophages^41^ and that *Mycobacterium abscessus* activates the NLRP3 inflammasome in a Dectin-1/Syk dependent manner^26^. In the present study we found that SEA induced NLRP3 inflammasome activation was similarly dependent upon Dectin-1/Syk.

Another kinase involved in NLRP3 inflammasome activation is JNK. JNK has been shown to be involved in the activity of ASC-containing inflammasomes in macrophages via a mechanism that involved the regulation of ASC speck formation. CPT-11, which has been shown to have great efficacy in anticancer therapy and is being used as the first line treatment for colorectal cancer, has recently been reported to activate the NLRP3 inflammasome through JNK signaling in macrophages^46^. The findings of the present study have suggested that SEA-induced NLRP3 inflammasome activation is dependent on JNK signaling.

In conclusion, we have demonstrated that the NLRP3 inflammasome can be activated in HSCs during *S. japonicum* infection via Dectin-1/Syk and JNK dependent signaling pathways. Our findings support a schistosomiasis-associated liver fibrosis model in which NLRP3 inflammasome activation in HSCs, then results in HSCs activation, collagen deposition and fibrosis. The data provide new insights into the pathogenesis of liver fibrosis during *S. japonicum* infection, and have identified novel molecular targets that could have potential in the development of therapies for the prevention and treatment of schistosomiasis-associated liver fibrosis.

## Methods

### Animals and reagents

BALB/c mice (4–6 weeks old, male, body weight 20 g) were obtained from a Schistosomiasis Control Laboratory in Hubei province, China. All animal experiments were in accordance with the National Institute of Health Guide for the Care and Use of Laboratory Animals, and were approved by the Committee on the Ethics of Animal Experiments of Tongji Medical College. The SSLF model was established by abdominal infection with *S. japonicum* cercariae according to the method used in the previous study^37, 47^. In brief, mice in the SSLF model group were percutaneously infected with *S. japonicum* by placing a glass slide carrying 20 ± 2 cercariae in nonchlorinated water in the abdomen for 15 min. Mice were then infected with adeno-associated virus AAV8-control or AAV8–shNLRP3 vectors at a dose of 5 × 10^9^ vg per animal, by tail vein injection (50μl total volume) using a 40 gauge ultra-fine insulin syringe. Mice in the control group were treated with nonchlorinated water containing no cercariae. All mice were raised for 6 weeks under pathogen-free conditions with free access to food and water.

*S. japonicum* egg antigen (SEA, 0.01 g/ml in phosphate-buffered saline (PBS)) was obtained from the Hubei Schistosomiasis Control Station. SEA was diluted to the working concentration in Dulbecco’s modified Eagle’s medium (DMEM) supplemented with 2% fetal bovine serum (FBS), before use.

### Isolation and culture of hepatic stellate cells (HSCs)

Infected mouse HSCs were prepared by the discontinuous density gradient centrifugation technique, as previously described^48^, with minor modifications^37^. The collected cells were cultured in DMEM containing 10% fetal bovine serum (FBS) (Gibco) in a humidified incubator at 37 °C with an atmosphere of 5% CO_2_. HSCs were characterized and confirmed as previously described^37, 47^. Cell viability, measured by the Trypan Blue exclusion assay, was determined to be approximately 90%. HSCs were treated with SEA (50 µg/ml) at the appropriate time according to the method. In certain experiments, HSCs were pre-treated for 1 h with either the Syk inhibitor piceatannol (200 µM (Abcam)), the JNK inhibitor SP600125 (20 µM (Abcam)), the p-38 inhibitor SB 203580 (20 µM (Abcam)) or Dectin-1 primary antibody (0.3 µg/ml) (R&D Systems), to inhibit the corresponding target.

### HE and Masson staining of liver tissues

Following anesthesia, the livers were excised, briefly rinsed in phosphate buffered saline, and then immediately placed in 4% (v/v) paraformaldehyde (PFA) in phosphate-buffered saline (PBS) at room temperature for 24 h. The liver specimens were embedded in paraffin, cut in to 5 µm sections, stained with hematoxylin and eosin (HE) to assess the extent of liver injury, and with Masson’s trichome stain to assess the level of collagen deposition. The data were represented by the area percentage of each slide that was positive for the blue stain, which was calculated in Image Pro Plus 6.0 software.

### Immunofluorescence staining

Cells seeded in 24-well chamber plates were washed three times, fixed in 4% paraformaldehyde, permeabilized with 0.1% Triton X-100 and incubated with a blocking solution containing 10% donkey serum and Phosphate-buffered saline containing 0.05% Tween-20. The cells were incubated with following primary antibodies: NLRP3 (1:50, Abcam), ASC (1:50, Santa Cruz), Caspase-1 (1:50, Santa Cruz), Syk (1:200, Abcam) overnight at 4 °C, washed three times and then with Alexa Fluor® 488 (1:200, Abcam) or Alexa Fluor® 555 (1:200, Abcam) 1 h at room temperature. Nuclei were stained with DAPI for 5 min. Staining was visualized through sequential scanning on an Olympus scanning confocal microscope (Olympus, Tokyo, Japan).

### Real-time PCR

Total RNA from HSCs was isolated using TRIZOL reagent (Invitrogen, Carisbad, CA, USA) according to the manufacturer’s instructions and quantified by a spectrophotometer. cDNA was synthesized from 0.5 µg total RNA by reverse transcription for 15 min at 37 °C and 5 s at 85 °C using the PrimeScript 1st Strand cDNA Synthesis Kit (TaKaRa, Japan). Equal amounts of the reverse transcriptional products were subjected to PCR amplification using SYBR Green as the fluorescence indicator on an AB iCycler system (AB, United States). Real-time PCR included initial denaturation at 95 °C for 30 s, followed by 40 cycles of 95 °C for 5 s, 60 °C for 30 s. The primers for mouse GAPDH were 5′-AGGTCGGTGTGAACGGATTTG-3′ forward and 5′-GGGGTCGTTGATGGCAACA-3′ reverse. The primers for mouse Syk were 5′-CTACCTGCTACGCCAGAGC-3′ forward and 5′-TTCCCTCTCGATGGTGTAGTG-3′ reverse. The primers for mouse NLRP3 were 5′-ATTACCCGCCCGAGAAAGG-3′ forward and 5′-CATGAGTGTGGCTAGATCCAAG-3′ reverse.

### Western blotting

Total proteins from HSCs and liver tissues were extracted using RIPA lysis buffer (50 mmol/L Tris, 150 mmol/L NaCl, 1% Triton X-100, 1% sodium deoxycholate, 0.1% SDS, at pH7.4) containing protease inhibitors cocktail. Proteins were denatured with 1 × SDS buffer and boiled for 10 min. Samples were run by 10% SDS-PAGE, transferred at 250 mA onto a PVDF membrane, and blocked using 5% nonfat dry milk in Tris-buffered saline with 0.1% Tween-20 (TBST). Then, the membranes were probed with following primary antibodies overnight at 4 °C against caspase-1 (1:200, Santa Cruz), NLRP3 (1:250, R&D), ASC (1:300, Santa Cruz), Syk (1:1000, Abcam), p-Syk (Y525 + Y526) (1:250, Abcam), p38 (1:1000, CST), p-p38 (1:1000, CST), JNK (1:1000, CST), p-JNK (Thr 183 + Tyr 185) (1:1000, CST), Cathepsin B (1:400, Abcam), GAPDH (1:10000). The membranes were then washed and incubated for 1 h with horseradish peroxidase (HRP) conjugated secondary antibody at 1:4000. The membranes were washed, and all blots were visualized on Kodak Omat X-ray film. The bands were quantitated in grayscale using Image J software (NIH, United States).

### RNA interference

HSCs were transfected with 50 nM small interfering RNA (siRNA) with Lipofectamine® 2000 transfection reagent (Invitrogen, Carisbad, CA, USA) according to the manufacturer’s protocol. RNA and protein expression of each target were examined by RT-PCR or Western blot to assess transfection efficiency of each siRNA. The sense small interfering RNA sequences were as follows: NLRP3–5′-GTACTTAAATCGTGAAACA-3′, Syk-5′-CCATCGAGAGGGAACTTAA-3′.

### Immunoprecipitation

Cell lysates were extracted using RIPA lysis buffer (comprising 50 mmol/L Tris, 150 mmol/L NaCl, 1% Triton X-100, 1% sodium deoxycholate, 0.1% SDS, at pH7.4) containing protease inhibitors cocktail. Cells lysates were pre-incubated for 30 min at 4 °C with Protein A/G PLUS-Agarose (Santa Cruz) and an appropriate control IgG. The cell lysates were then centrifuged at 2,500 rpm for 5 min at 4 °C, before transferring an equal amount of protein to a new 1.5 ml microcentrifuge tube and incubating with ASC (Santa Cruz) or caspase-1 (Santa Cruz.) primary antibody for 1 h at 4 °C. Subsequently, the cell lysates were incubated with Protein A/G PLUS-Agarose (Santa Cruz) at 4 °C overnight. The protein A/G PLUS-Agarose was then collected and washed three times with cold lysis buffer. Laemmli’s sample buffer (30 μl) was added and the resultant mixture boiled for 5 minutes. After boiling, the samples were centrifuged at 2,500 rpm for 5 min to discard the Protein A/G PLUS-Agarose. Samples were then loaded on to a 10% acrylamide gel and subjected to gel electrophoresis.

### ELISA assay of IL-1β

Cell supernatant was harvested and IL-1β levels were detected using commercial ELISA kit (BioLegend, San Diego, USA) according to the manufacturer’s instructions.

### Statistical analysis

Data are expressed as means ± SEM. The statistical analysis was performed using GraphPad Prism 5.0 Software. The student’s test was used to evaluate the differences between two groups. One-way ANOVA was used to evaluate all other differences among multiple groups of data. P < 0.05 was considered statistically significant.

## Electronic supplementary material

Supplementary Information
