# Supplementary material for: NLRP3 inflammasome activation results in liver inflammation and fibrosis in mice infected with Schistosoma japonicum in a Syk-dependent manner
Source: Sci Rep. 2017 Aug 14;7:8120. doi: 10.1038/s41598-017-08689-1 (PMC5556086; doi:10.1038/s41598-017-08689-1)
Supplement: Supplementary file 1 — Supplementary Information [file 41598_2017_8689_MOESM1_ESM.doc]

Supplementary data to:

**NLRP3 inflammasome activation results in liver inflammation and fibrosis in mice infected with *Schistosoma japonicum* in a Syk-dependent manner**

Ya-Qi Lu^1^, Shan Zhong^1^, Nan Meng^1^, Yin-ping Fan^1^, Wang-Xian Tang^1,*^

^1^Institute of Liver Diseases, Tongji Hospital of Tongji Medical College, Huazhong University of Science and Technology, 1095 Jie-Fang Avenue, Wuhan 430030, Hubei Province, People’s Republic of China

**Supplementary Figures**

**Supplementary Fig.S1.** Full-length blots of Fig.1b are included in the Supplementary Information file.


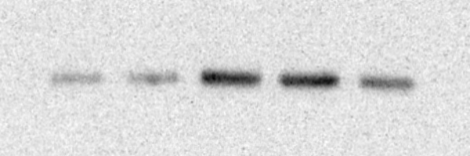
 NLRP3


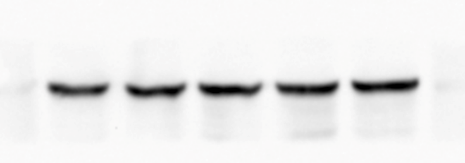
 GAPDH

**Supplementary Fig.S2.** Full-length blots of Fig.2d are included in the Supplementary Information file.


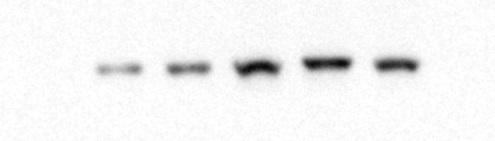
 collagen-1


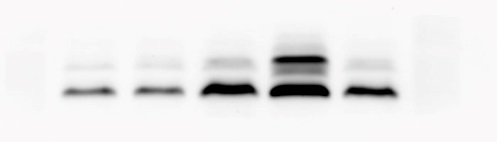
 TIMP-1


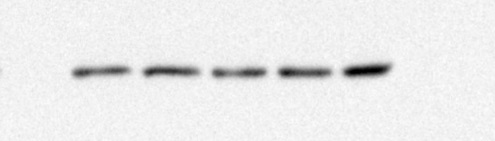
 GAPDH

**Supplementary Fig.S3.** Full-length blots of Fig.4a are included in the Supplementary Information file.


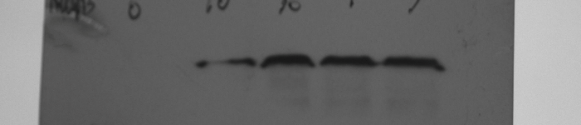
 NLRP3


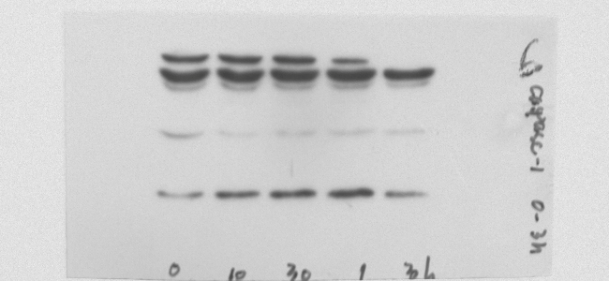
 procaspase-1/caspase-1


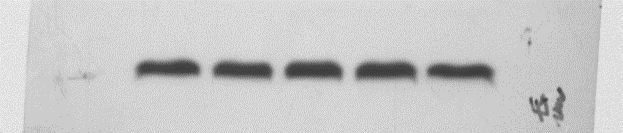
 GAPDH

**Supplementary Fig.S4.** Full-length blots of Fig.4e are included in the Supplementary Information file.


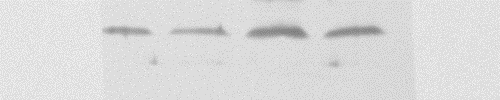
 NLRP3


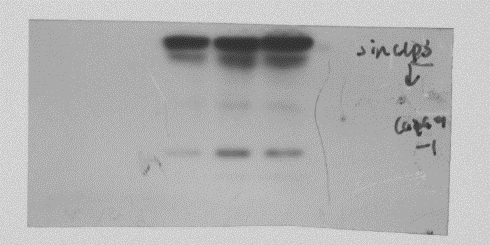
 procaspase-1/caspase-1


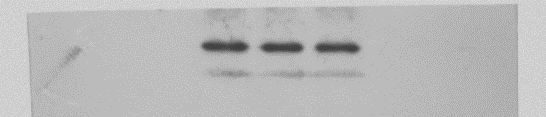
 GAPDH

**Supplementary Fig.S5.** Full-length blots of Fig.5a are included in the Supplementary Information file.


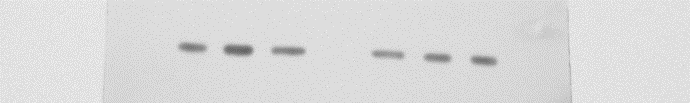
 collagen-1


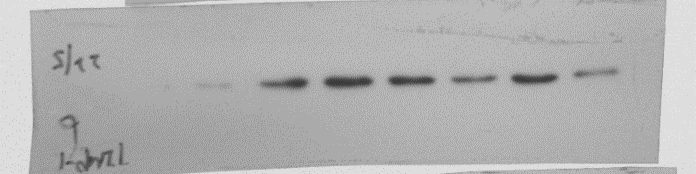
 TIMP-1


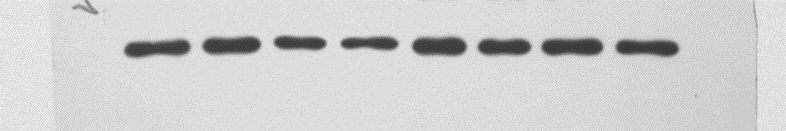
 GAPDH

**Supplementary Fig.S6.** Full-length blots of Fig.6a are included in the Supplementary Information file.


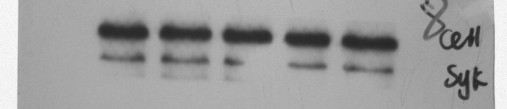
Syk


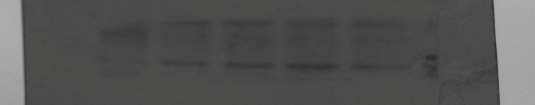
 p-Syk


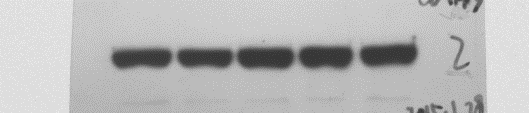
 GAPDH

**Supplementary Fig.S7.** Full-length blots of Fig.6c are included in the Supplementary Information file.

IP: ASC


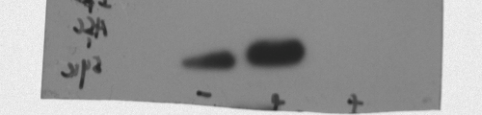
 Syk


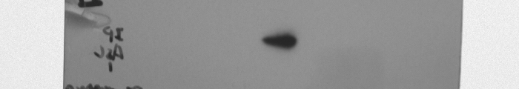
 procaspase-1


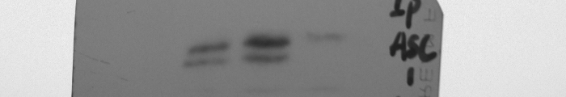
 ASC


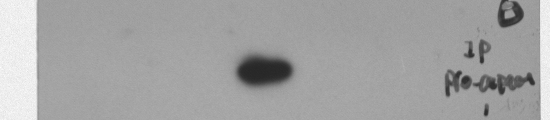
 NLRP3

Input: 0-1h


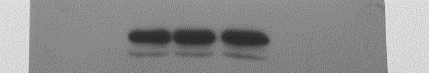
 Syk


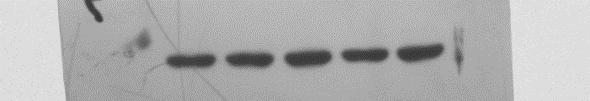
 procaspase-1


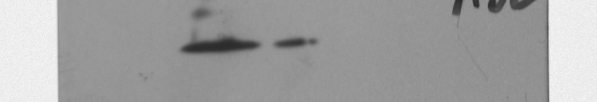
 ASC


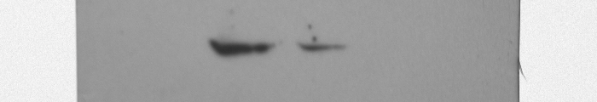
 NLRP3

IP: caspase-1


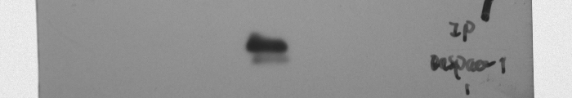
 Syk


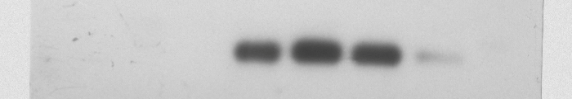
 procaspase-1


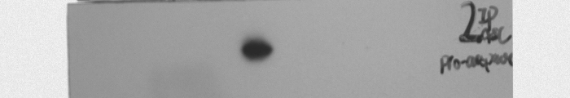
 ASC


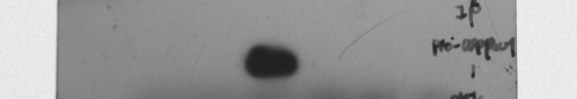
 NLRP3

**Supplementary Fig.S8.** Full-length blots of Fig.6e are included in the Supplementary Information file.


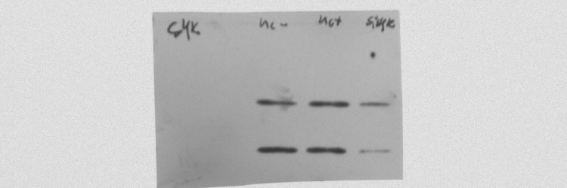
 Syk


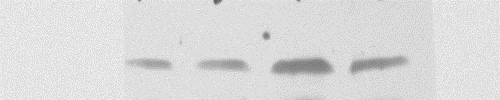
 Cathepsin B


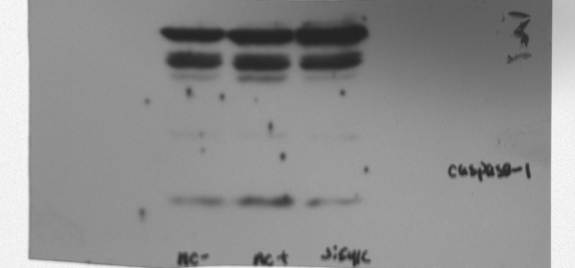
 procaspase-1/caspase-1


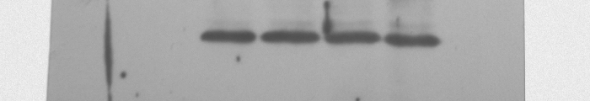
 GAPDH

**Supplementary Fig.S9.** Inhibiting Syk by piceatannol reduces NLRP3 inflammasome activation.


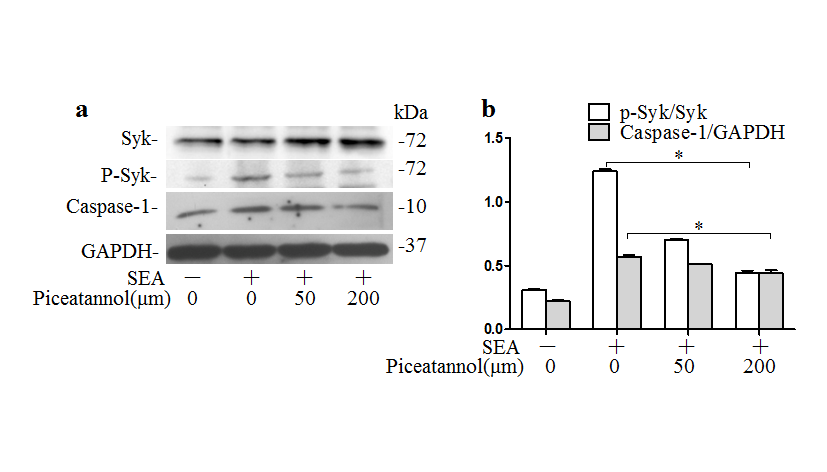


**Fig.S9.** Inhibiting Syk by piceatannol reduces NLRP3 inflammasome activation: HSCs were incubated with or without SEA (50 μg/ml) for 2 h in the presence or absence Syk inhibitor piceatannol, (a) western blot gels and (b) summarized data showing the expression of Syk, p-Syk (Y525/526), activated caspase-1 and GAPDH, full-length blots are included in the supplementary Fig.S10. Results are a representative of at least three independent experiments. *p﹤0.05.

**Supplementary Fig.S10.** Full-length blots of Fig.S9a are included in the Supplementary

Information file.


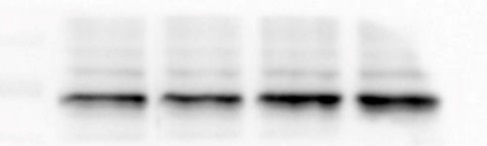
 Syk


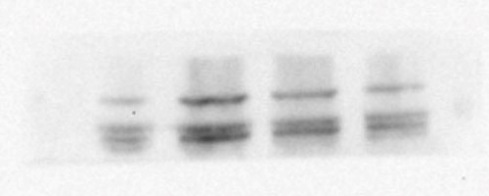
 p-Syk


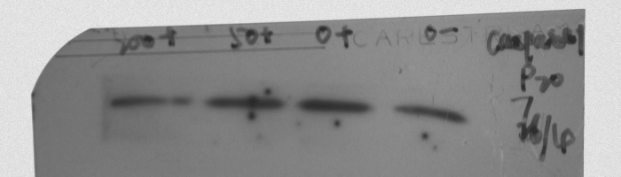
 caspase-1


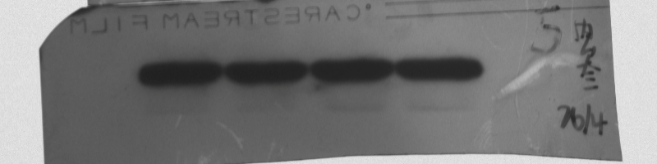
 GAPDH

**Supplementary Fig.S11.** Full-length blots of Fig.7a are included in the Supplementary Information file.


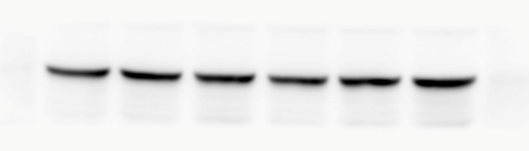
 Syk


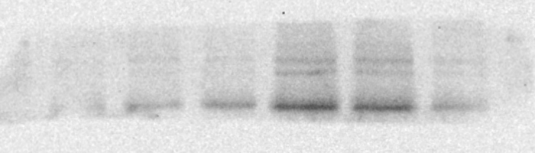
 p-Syk


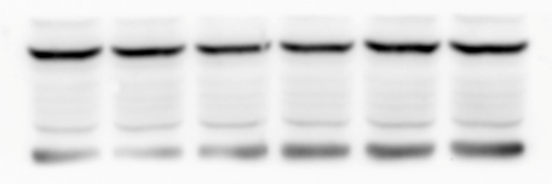
Pro-caspase-1


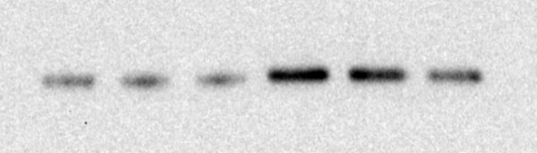
Caspase-1


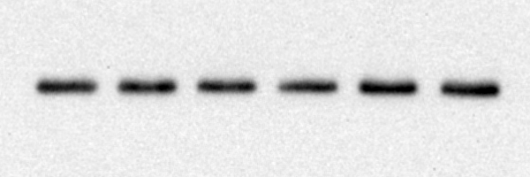
 GAPDH

**Supplementary Fig.S12.** Full-length blots of Fig.7d are included in the Supplementary Information file.


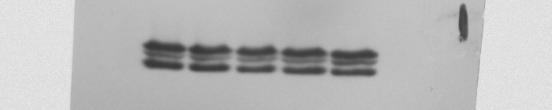
 JNK


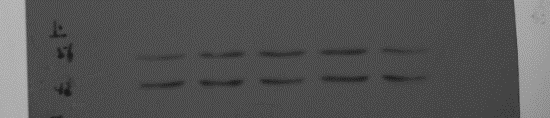
 p-JNK


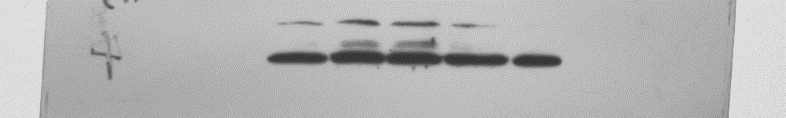
 p38


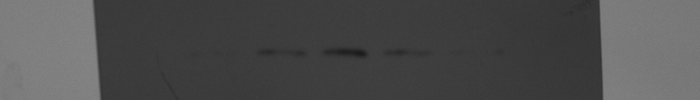
 p-p38


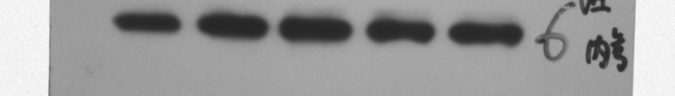
 GAPDH

**Supplementary Fig.S13.** Full-length blots of Fig.7f are included in the Supplementary Information file.


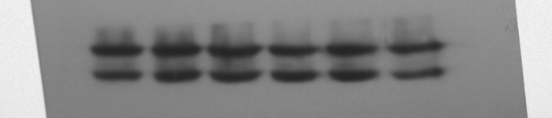
 JNK


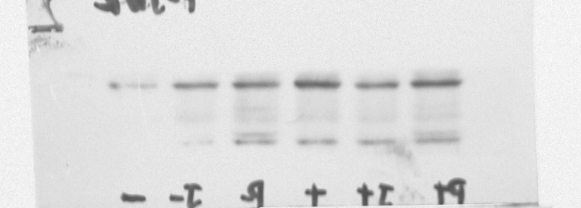
 p-JNK


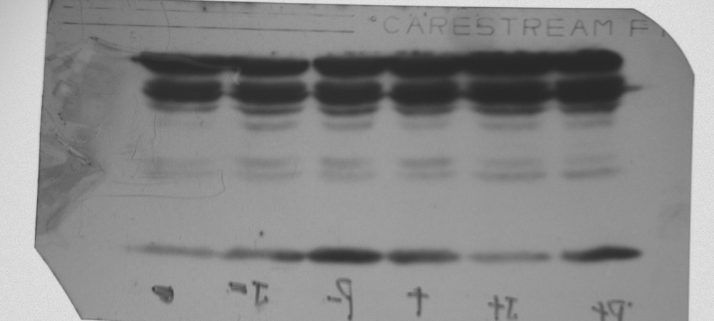
 procaspase-1/caspase-1


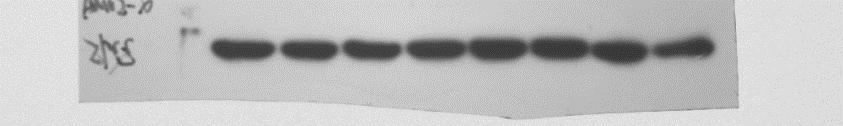
 GAPD
